# Factors Influencing Implementation of Blood Transfusion Recommendations in Pediatric Critical Care Units

**DOI:** 10.3389/fped.2021.800461

**Published:** 2021-12-17

**Authors:** Katherine M. Steffen, Philip C. Spinella, Laura M. Holdsworth, Mackenzie A. Ford, Grace M. Lee, Steven M. Asch, Enola K. Proctor, Allan Doctor

**Affiliations:** ^1^Division of Pediatric Critical Care Medicine, Department of Pediatrics, Stanford University, Palo Alto, CA, United States; ^2^Division of Pediatric Critical Care Medicine, Department of Pediatrics, Washington University in Saint Louis, Saint Louis, MO, United States; ^3^Department of Medicine, Primary Care and Population Health, Stanford University, Stanford, CA, United States; ^4^Division of Pediatric Cardiology, Department of Pediatrics, Stanford University, Palo Alto, CA, United States; ^5^Division of Pediatric Infectious Diseases, Department of Pediatrics, Stanford University, Stanford, CA, United States; ^6^Department of Medicine, Primary Care and Population Health, Stanford University, Stanford, CA, United States; ^7^George Warren Brown School of Social Work, Washington University in St. Louis, St. Louis, MO, United States; ^8^Division of Pediatric Critical Care Medicine, Department of Pediatrics, University of Maryland, Baltimore, MD, United States

**Keywords:** erythrocyte transfusion, critical care, pediatrics, pediatric intensive care unit, clinical practice guideline, implementation science

## Abstract

**Purpose:** Risks of red blood cell transfusion may outweigh benefits for many patients in Pediatric Intensive Care Units (PICUs). The Transfusion and Anemia eXpertise Initiative (TAXI) recommendations seek to limit unnecessary and potentially harmful transfusions, but use has been variable. We sought to identify barriers and facilitators to using the TAXI recommendations to inform implementation efforts.

**Materials and Methods:** The integrated Promoting Action on Research Implementation in Health Services (iPARIHS) framework guided semi-structured interviews conducted in 8 U.S. ICUs; 50 providers in multiple ICU roles completed interviews. Adapted Framework analysis, a form of content analysis, used the iPARIHS innovation, recipient, context and facilitation constructs and subconstructs to categorize data and identify patterns as well as unique informative statements.

**Results:** Providers perceived that the TAXI recommendations would reduce transfusion rates and practice variability, but adoption faced challenges posed by attitudes around transfusion and care in busy and complex units. Development of widespread buy-in and inclusion in implementation, integration into workflow, designating committed champions, and monitoring outcomes data were expected to enhance implementation.

**Conclusions:** Targeted activities to create buy-in, educate, and plan for use are necessary for TAXI implementation. Recognition of contextual challenges posed by the PICU environment and an approach that adjusts for barriers may optimize adoption.

## Introduction

Red blood cell (RBC) transfusions can be lifesaving when administered for hemorrhagic shock or severe anemia; however, mounting evidence suggests that unnecessary transfusions have more risks than benefits (1–5). In critically ill patients in particular, the benefit of transfusion may only be noted when normal physiologic compensation for anemia is compromised or has failed. Recent data suggests that donor RBCs are inferior to native cells in delivering oxygen, may have negative pro-coagulant and immunomodulatory effects (6–9), and increase risk for both infectious and non-infectious serious hazards of transfusion (10–12). Transfusion has been independently associated with an increased duration of mechanical ventilatory support, increased risk of multi-organ system dysfunction and death (13, 14). Restrictive transfusion strategies significantly reduce exposure to RBCs without negatively impacting clinical outcomes (15–25). The Transfusion and Anemia eXpertise Initiative (TAXI) developed evidence-based and expert-informed recommendations to limit unnecessary and potentially harmful RBC transfusions in critically ill children (26). The TAXI group took an innovative approach to include considerations for implementation during recommendation development. The main publication featured a decision tree to summarize the recommendations for easy use. Despite the evidence and publication of these recommendations, multiple studies have documented only partial adoption of restrictive transfusion strategies (14, 27, 28), exposing children to transfusion risks without benefit (9, 14, 27–30). Additional efforts are required to integrate these recommendations into practice in pediatric intensive care units (PICUs).

We utilized an established implementation framework, the integrated Promoting Action on Research Implementation in Health Services (iPARIHS) framework (31), to explore barriers and facilitators to implementing the TAXI recommendations in the PICU. The framework conceptualizes three constructs: the innovation, recipients, and context, which are modified by a fourth construct, facilitation. The iPARIHS explains factors necessary for implementation and acknowledges that implementation is both complex and dynamic in practice (31). In this qualitative study, we sought to identify elements that influence use of the TAXI recommendations in anticipation of formal implementation.

## Materials and Methods

### Study Design

Semi-structured qualitative interviews were carried out to assess prospective implementation of the TAXI recommendations, including identifying barriers and facilitators to implementation in different PICU settings. Ethics approval for the study was obtained from the Stanford University Institutional Review Board (IRB-47140).

### Participants and Setting

We conducted interviews with health care providers working in various roles from eight PICUs across the United States (US). Units represented known variation in PICU types (pediatric ICU excluding cardiac patients, 4 units), pediatric cardiovascular ICU (CVICU) (2 units) and combined PICU/CVICU (2 units) and overall size (11–32 beds) and geographic location. Provider types were selected based on anticipated familiarity with and influence on RBC transfusion in the ICU and included ICU attendings, fellow and resident trainees, nurse practitioners (NPs), nurses, and subspecialty physicians/surgeons whose patients were cared for regularly in pediatric ICUs including hematologists/oncologists, cardiologists, and general and cardiothoracic surgeons. A unit representative helped recruit participants via email. The study team provided additional information to interested respondents. We selected participants using a stratified, purposeful sampling strategy (32) to achieve a sample of participants within each PICU role. We developed an interview guide based on iPARIHS (31) to examine barriers and facilitators to using the TAXI recommendations in the ICU (Supplementary File 1); interviews covered all topics, however interviewers asked follow-up questions to explore topics fully. Additional topics included transfusion decision making and general perceptions around implementing clinical practice changes; data related to these topics are reported elsewhere (33). One of two authors (KS and MF) conducted interviews either in person or via telephone after obtaining verbal consent. Interviews were audio recorded and transcribed verbatim. We interviewed participants in each role until no new information was forthcoming and data saturation was achieved. Study team members (KS, LH, and GL) reviewed data to confirm when variation in responses was no longer noted.

### Qualitative Analysis

We used the Framework Approach (34) for qualitative analysis with QSR International's NVivo (Version 12) software. This approach summarizes case-level data (rows) along thematic categories (columns) in a matrix and involves: familiarization, identifying a thematic framework, indexing, charting, and mapping and interpretation (35). Coding was structured around the iPARIHS innovation, recipients, context, and facilitation constructs and subconstructs (31) to gain insight into the combination of factors to better specify and tailor strategies for successful implementation (36, 37). Innovation consists of the evidence implemented and alignment of the evidence with local priorities and practice. The recipients construct acknowledges the impact individuals or teams may have on supporting or resisting innovation. Context is defined as the “environment or setting in which the proposed change is to be implemented.” iPARIHS facilitation involves “activating implementation through assessing and responding to characteristics of the innovation and recipients within their contextual setting” (31). We developed a codebook to standardize subconstruct definitions for analysis (Supplementary File 2) based on other common implementation framework definitions (38–40). Two researchers (KS, LH) coded five interviews independently using a priori codes from iPARIHS while creating inductive codes for emergent themes. One researcher (KS) coded the remainder of the interviews using the established strategy. The researchers met to agree on definitions and interpretations of existing codes, compare coding, and discuss emerging themes. Following summarization of coded data, we compared and contrasted data between roles and ICUs, identifying patterns and unique, but informative statements to capture variation. Strategies to ensure internal validity followed guidance by Miles et al. (41) and included linking data to iPARIHS categories, checking for negative evidence, and checking that findings were replicable across more than one ICU.

## Results

**Figure 1:**
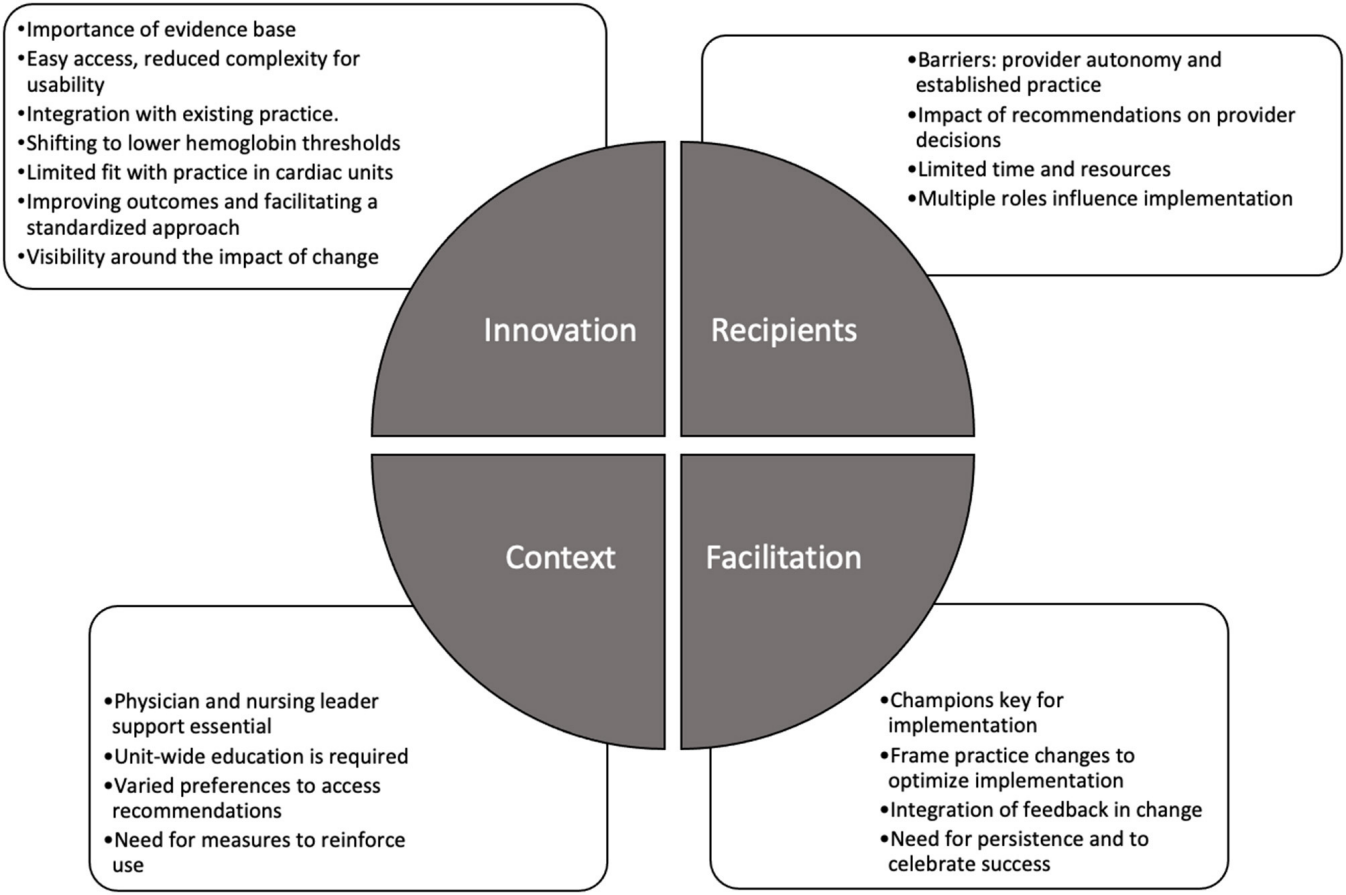
Themes identified within each of the iPARIHS constructs.

Fifty health care providers were interviewed: ICU attendings (*n* = 15, median duration of experience in role = 6 years), fellow (6, 1.5 years), and resident (4, 1.75 years) trainees, NPs (9, 5 years), nurses (10, 5 years), and subspecialty physicians/surgeons (6, 4.5 years). Of the attending providers, 8 worked in PICUs, 4 worked in CVICUs and 3 worked in combined PICU/CVICUs. Providers in each role were interviewed at each site, with the exception of fellow or resident trainees, who were not present in all units. Unless specified, “providers” refers to these roles collectively. Interviews lasted a median of 53.5 min (range = 37–79 min) and were completed between December 2018 and June 2019. We identified 19 themes across the four iPARIHS constructs; themes are presented below under each of the four constructs (Figure 1). The innovation construct provided insights into use of the recommendations, while the recipients, context, and facilitation constructs informed potential implementation approaches.

### iPARIHS Innovation

The Innovation construct included themes such as the quality of the evidence and the degree of fit with existing practice and values (31). Providers were generally supportive of clinical practice recommendations, stating that they improved knowledge, use of evidence-based practice, and were useful to frame thinking in ambiguous clinical situations, even when based on expert opinion.

#### Critical Importance of the Evidence Base

Providers in all roles indicated that a strong evidence base was influential in their decision to incorporate new practice changes. The evidence-based nature of the TAXI recommendations had value in influencing other providers: “*For those people who are a little bit more set in their ways and may not want to listen, having that evidence would certainly be convincing*” (Site 1 CVICU, Attending 1). Differences in how providers viewed evidence based on unit-type and role are detailed in Tables 1, 2.

**Table 1:** Differences in perceptions of the iPARIHS innovation construct by critical care setting.

**Themes related to the iPARIHS Innovation Construct**	**PICU**	**CVICU**	**Combined PICU/CVICU**
Critical importance of the evidence base	TAXI recommendations could be easily accepted by providers	Transfusion thresholds for CVICU patients disparate from current practice, harder to support given lack of larger studies in sub-populations (i.e., single ventricle patients) Transfusion thresholds may need to be increased for Stage 1 single ventricle repairs to be accepted	Transfusion thresholds for CVICU patients disparate from current practice, harder to support given lack of larger studies in sub-populations (i.e., single ventricle patients) Transfusion thresholds may need to be increased for Stage 1 single ventricle repairs to be accepted Cardiothoracic surgeons may also not accept thresholds for Stage 2 and 3 single ventricle repairs
Ease of access and reducing complexity to enhance usability	Units without some services (without ECMO, dialysis, cardiac patients) preferred simplified decision tree that omitted these populations, services	Preference to see decision tree recommendations specific to cardiac patients only	Entire decision tree necessary to care for all patients
	Converting hemoglobin to hematocrit thresholds helpful in units more familiar with discussing hematocrit	Converting hemoglobin to hematocrit thresholds helpful in units more familiar with discussing hematocrit	Converting hemoglobin to hematocrit thresholds helpful in units more familiar with discussing hematocrit

*iPARIHS, integrated Promoting Action on Research Implementation in Health Services; PICU, Pediatric Intensive Care Unit; CVICU, Cardiovascular Intensive Care Unit; TAXI, Transfusion and Anemia eXpertise Initiative; ECMO, Extracorporeal Membrane Oxygenation*.

**Table 2:** Differences in iPARIHS constructs by professional role.

**iPARIHS construct and themes related to that construct**	**Attending/ fellow**	**Nurse Practitioner**	**Nurse**
Innovation-Critical importance of the evidence base	Preferred research-based evidence as primary justification for practice change Barrier to TAXI recommendation use: lack of strong evidence around many recommendations Appreciated TAXI recommendations indicated where strong evidence was lacking, when clinical judgment necessary	Appreciated TAXI recommendations indicated where strong evidence was lacking, when clinical judgment necessary	Expressed wanting to understand rationale for practice change, however strong research-based evidence supporting change not always necessary
Innovation-Shifts in transfusion practice	No data	Hesitancy using recommendations when hemoglobin 5–7 g/dL, with a desire to see additional evidence around specific impact on patient outcomes, possible unintended adverse consequences	Hesitancy using recommendations when hemoglobin 5–7 g/dL, with a desire to see additional evidence around specific impact on patient outcomes, possible unintended adverse consequences
Innovation-Need for visibility around the impact of change	Regular individual or unit-level feedback on compliance and outcomes was important to increase buy-in and sustainability	Concerned about unintended consequences of recommendation compliance, expressed interest in monitoring this data.	Concerned about unintended consequences of recommendation compliance, expressed interest in monitoring this data.
Recipient-Multiple roles influence implementation of the transfusion recommendations	Physician resistance was anticipated to be one of the critical barriers to recommendation use ICU attendings anticipated difficulty obtaining consensus around transfusion thresholds among subspecialists and surgeons, particularly cardiac surgeons	NPs were a consistent presence in the unit and could influence trainees by directing them to the recommendations as standard unit practice	The opinion of nurses was also highly valued: “*A lot of experienced nurses will bring up transfusion as a treatment. And I put a lot of stock in our nurses' opinion because they're tremendous and have a ton of experience. So, they have a big role to play in our transfusion practices*” (CVICU 1, Attending 1)
Context-Variation in provider educational preferences	Important to review evidence supporting recommendations at didactic conferences, journal clubs, other meetings Fellows noted hearing attending perspectives was important to shape their practice	No data	Multi-modal educational opportunities needed to address different learning preferences Presenting recommendations at nursing council, staff meetings, or seminars recommended. Dissemination in printed materials (newsletters, flyers, posters) also recommended
			Education more effective if provided proximal to “go-live” date, with practical application examples
			Nursing educators and managers essential to inform and remind nurses about changes, their role in change
Facilitation-Framing change to align with objectives and ideals	Some attending providers valued changes aligned with the hospital's mission or that elevated group practice to be consistent with other top institutions	No data	No data

*iPARIHS, integrated Promoting Action on Research Implementation in Health Services; TAXI, Transfusion and Anemia eXpertise Initiative; g/dL, grams per deciliter; NP, Nurse Practitioner; ICU, Intensive Care Unit; CVICU, Cardiovascular Intensive Care Unit*.

#### Ease of Access and Reducing Complexity to Enhance Usability

Given the complexity of the recommendations, easy access to the TAXI decision tree and reference materials were essential. Variability in provider preferences around decision tree visualization based on unit type are detailed in Table 1.

#### Optimizing Integration With Existing Practice

Practice changes were more consistently supported and adopted when there was strong consensus around change, when changes were integrated into existing workflows, or when changes improved work processes.

#### Shifts in Transfusion Practice to Lower Hemoglobin (Hb) Thresholds

Reported wide variation in practice and regular transfusions above TAXI Hb thresholds highlighted opportunities to reduce unnecessary transfusions. Some providers found the recommendations restrictive, while others found them to be consistent with their current practice. Acceptability varied based on ICU role (Table 2). Providers identified that the TAXI recommendations would alter their approach to transfusion most for patients with a Hb of 5–7 g/dL, where transfusion can be considered, but not automatically given. For some, the recommendations validated the decision to monitor an anemic patient.

#### Limited Degree of Fit With Existing Practice in Units Caring for Cardiac Patients

Many PICU providers felt the TAXI recommendations could be readily accepted, whereas gaps between the TAXI-recommended Hb thresholds and current practice existed for cardiac patients, making it difficult for providers who cared for these patients to accept the recommendations. Information around TAXI implementation in single ventricle patients is detailed in Table 1.

#### Improving Patient Outcomes and Facilitating a Standardized Approach

Providers noted potential benefits of using the TAXI recommendations including reducing rates of transfusion, blood product exposure, and transfusion complications. Providers felt the recommendations facilitated an organized approach to decision making and a standard unit-wide approach across groups with different backgrounds. Attending physicians indicated that the recommendations could aid in transfusion discussions with nurses and families and when providers disagreed. The recommendations were seen as a resource for less experienced providers to increase confidence in decision making and discussing transfusion.

#### Need for Visibility Around the Impact of Change

Providers noted that the TAXI recommendations would be more easily accepted if they improved patient outcomes, care processes, or patient safety. While providers noted that disseminating process and outcomes data aided adoption, infrastructure to provide these data was lacking in most institutions. Role-specific information around visibility of data is noted in Table 2.

### iPARIHS Recipients

Themes included the recipients construct included provider knowledge, limited time and resources, and the influence of multiple provider roles on transfusion decisions.

#### Provider Autonomy and Established Practice as Barriers to Acceptance of Recommendations

While many physicians voiced willingness to use the TAXI recommendations themselves, CVICU and CVICU-PICU providers indicated that some attendings were “more set in their ways” and unlikely to follow the recommendations. Potential reasons for this resistance included preconceived beliefs about transfusion risks and benefits and the opinion that the recommendations were not applicable to the provider's patient population.

#### Impact of the Transfusion Recommendations on Provider Decisions

Providers indicated that the TAXI recommendations would be useful for individuals who were not updated on current literature. Some providers recognized that the recommendations require clinical judgement, and that guidance for trainees and others with less experience may be required: “*understanding what clinical judgment “is” is clear [to me] but defining what clinical features go into that decision might be helpful for trainees*” (Site 3 PICU, NP 23).

#### Limited Time and Resources Impact Transfusion Practice

Monitoring anemia was more difficult when the ICU was busy, full, or understaffed, and resulted in earlier transfusion to avoid complications related to anemia. One nurse noted that physicians “*don't want the patient's hemoglobin to drop too low and they're off somewhere else, so they want a cushion*” (Site 1 Combined PICU/CVICU, Nurse 13). A few providers noted that it required more effort to monitor an anemic patient, and transfusion obviated the need to follow laboratory studies as closely. Heavy workloads and frequent clinical practice changes were challenges to consistent recommendation use: “*There's so many checks and things that you have to do, you just don't have time. It's one more thing that we have to do*.” (Site 2 Combined PICU/CVICU, Nurse 48).

#### Multiple Roles Influential in Implementing Transfusion Recommendations

Providers in each ICU role had an impact on transfusion decision making, underscoring the importance of achieving buy-in and alignment around using the recommendations. Table 2 details the impact of each role type.

### iPARIHS Context

For implementation of the TAXI recommendations, themes within the context construct included the role of unit leaders and methods to educate, disseminate, and provide access to the recommendations.

#### Physician and Nursing Leader Support Is Essential

While multi-professional buy-in around using recommendations was necessary, ensuring formal support from ICU physician and nursing leaders was also vital for adoption, with a few providers noting that support of the nurse educator was also important.

#### Unit-Wide Education Is Required

Providers expressed interest in learning about the TAXI evidence base, but learning preferences differed based on provider role (see Table 2). Multi-professional education was needed to facilitate support across the ICU team: “*Even if the nurses aren't [ordering] a transfusion, they're more likely to follow [recommendations] and feel comfortable with not transfusing if they understand why*” (Site 4 PICU, NP 37). Clear explanations around the rationale and goals of a change enhanced provider acceptance, along with tips to integrate the change into existing workflows.

#### Accessing the Recommendations: Varied Preferences for Formatting and Location

Access to the recommendations was essential while making transfusion decisions, reviewing labs, and during rounds. No single tool or method to access the recommendations was favored, indicating that local tailoring is required. Suggested Electronic Medical Record (EMR)-based tools included links to reference materials or full integration into transfusion orders. One provider noted that the individual ordering a transfusion often did not make the ultimate decision to transfuse, reducing the potential impact of EMR-based order sets.

#### Methods to Reinforce Use of the Recommendations

Regular use of new practices aided in integration into unit workflow. EMR-based alerts when an un-indicated transfusion was ordered and reminders in daily huddles, daily discussions about transfusion thresholds during rounds or sign out, or as part of a safety checklist were other potential mechanisms to ensure habitual use.

### iPARIHS Facilitation

Data elements coded under facilitation often were linked with one or more of the other iPARIHS constructs and have been described above, including the need for widespread alignment and buy-in, multiple educational modalities and easy access to reference materials. The following themes were unique to the facilitation construct.

#### Champions as Key Facilitators of Implementation

Champions were identified as highly influential in successful implementation. A champion was defined by one provider as: “*an ever- present voice, making sure that there's a steady flow of reminders that “this is what we're doing. This is why we're doing it. It's important”*” (Site 2 PICU, Hematologist-Oncologist 18). A committed TAXI champion from each professional role was felt to be useful to support others in that role.

#### Keys to Framing Practice Changes to Optimize Implementation

Changes that were consistent with unit principles and goals were adopted more easily. Thus, framing changes in a way that aligns with common objectives and ideals may aid in implementation (see Table 2 for role-specific details).

#### Integration of Feedback in Planning and Operationalization of Change

Providers commonly valued when their feedback was solicited and integrated into implementation plans. Engagement of the entire multi-professional PICU team was a motivator to support and adopt changes. Ongoing solicitation and integration of feedback optimized sustainability over time.

#### Need for Persistence and Celebrating Successes

Persistence and consistency in promoting and using a change were critical. Acknowledgment and celebration when a change was used successfully helped to recognize provider efforts, demonstrate benefits to late adopters, and promote sustainability.

## Discussion

While the TAXI recommendations provide evidence-based guidelines for transfusion, little is known about how to implement them in routine practice. The objective of this study was to use the iPARIHS framework to explore barriers and facilitators to inform development of targeted implementation strategies to achieve this goal. This effort represents a first step in reducing unnecessary and potentially harmful transfusions in critically ill children and may also inform implementation of other evidence-based recommendations in the pediatric critical care setting.

Our analysis highlights the complexity of the PICU and importance of a team-based implementation approach. The quality of evidence impacted willingness to adopt the TAXI recommendations and underscored the need for guidance for less experienced providers in applying clinical judgement. While transfusion practices were noted to vary and thus benefit from a standardized approach, the recommendations were anticipated to alter practice most in (1) patients with a Hb 5–7 g/dL and (2) hemodynamically stable single ventricle patients. Acceptance of the recommendations for single ventricle patients posed the greatest challenge to provider acceptance.

Multi-professional support and widespread education was needed, with acknowledgment that many roles influence transfusion decision making. Larger units may face greater challenges ensuring dissemination of educational materials and consistent uptake of the recommendations than smaller units, though a robust and multi-faceted implementation approach may be useful. Providers viewed the TAXI recommendations as a tool to educate around restrictive transfusion and justify the decision to defer transfusion. This study examines aspects of ICU transfusion practice that have not been previously addressed. Adult studies have reported lack of physician knowledge about transfusion risks and indications (42), and lack of confidence in using restrictive approaches (43, 44). This study reveals that decision-making in pediatric ICUs is influenced by multiple provider roles and highlights the importance of building consensus and a team-based approach to implementation. Fortin et al. found that pediatric providers received little academic training in transfusion and learned through practice or from colleagues (45). Dismantling learned practices may be difficult but providing education around evidence-based recommendations may be key to improving and standardizing practice.

This study informs potential implementation of the TAXI recommendations, but also highlights themes that are likely important for any implementation effort in the PICU: (1) the need for multi-professional involvement in buy-in, implementation planning, and education, (2) appreciation of the complexity of care and competing demands, which drive the need for committed and persistent champions, and (3) the need for resources to support implementation and monitoring of implementation and clinical outcomes (46). High-acuity and high-stakes care as well as interaction with multiple provider teams and subspecialty groups increase complexity of working in the ICU. In many cases, multiple active initiatives to improve care or otherwise modify existing workflow may further complicate care. Committed champions can create support for specific initiatives and re-focus efforts among individual provider groups when necessary. Notably, resources for monitoring outcomes data were insufficient in most units yet felt to be important for enacting change.

Limitations of this study include the fact that a relatively small number of providers in each role were interviewed, which may account for heterogeneity of some responses. We did not include hospital leaders or transfusion directors, who were outside the scope. These individuals were often noted to be supporters of standardized, evidence-based recommendations such as TAXI. Providers volunteered to participate in this study and their perspectives may not be representative of all individuals. Units were selected to represent the scope of US practice; however, the data may not be fully generalizable to other PICUs, although using the iPARIHS framework helped to ensure that we captured implementation characteristics that could be broadly transferable across settings. While this analysis may not reflect all barriers, it yields information that serves as a starting point to guide implementation in this setting.

## Conclusion

Individuals in multiple PICU roles influence transfusion decision making. Implementing the TAXI recommendations will likely require strategies targeted to create buy-in, plan for use, and educate providers. Integration of the recommendations into existing workflow and committed champions to promote use despite challenges posed by the complex and busy environment are important, along with providing resources to implement and monitor the impact on patient outcomes. A structured implementation approach that adjusts for known barriers will optimize adoption and may aid in implementation of other evidence-based recommendations.

## Data Availability Statement

The raw data supporting the conclusions of this article will be made available by the authors, without undue reservation.

## Ethics Statement

The studies involving human participants were reviewed and approved by Stanford University Institutional Review Board - IRB 47140. Written informed consent for participation was not required for this study in accordance with the national legislation and the institutional requirements.

## Author Contributions

KS conceptualized the study, curated data, developed the study methodology, contributed to the completion of the investigation and formal analysis, wrote the original draft, and reviewed and edited this manuscript. LH conceptualized the study, curated data, developed the study methodology, contributed to the formal analysis, and reviewed and edited this manuscript. MF aided in the execution of the investigation and reviewed and revised the manuscript. AD, GL, and PS helped conceptualize the study, reviewed the formal data analysis, and reviewed and edited this manuscript. EP and SA contributed to the conceptualization of the study and reviewed and edited this manuscript. All authors approved the final manuscript as submitted and agreed to be accountable for all aspects of the work.

## Funding

Research reported in this publication was supported by the National Heart, Lung, and Blood Institute of the National Institutes of Health [K12HL137942].

## Author Disclaimer

The content is solely the responsibility of the authors and does not necessarily represent the official view of the National Institutes of Health.

## Conflict of Interest

The authors declare that the research was conducted in the absence of any commercial or financial relationships that could be construed as a potential conflict of interest.

## Publisher's Note

All claims expressed in this article are solely those of the authors and do not necessarily represent those of their affiliated organizations, or those of the publisher, the editors and the reviewers. Any product that may be evaluated in this article, or claim that may be made by its manufacturer, is not guaranteed or endorsed by the publisher.

## Abbreviations

CVICU: Cardiovascular Intensive Care Unit
ECMO: Extracorporeal Membrane Oxygenation
EMR: Electronic Medical Record
Hb: Hemoglobin
iPARIHS: integrated Promoting Action on Research Implementation in Health Services
NP: nurse practitioner
PICU: Pediatric Intensive Care Unit
RBC: Red Blood Cell
TAXI: Transfusion and Anemia eXpertise Initiative
US: United States.
