# Supplementary material for: Factors Influencing Implementation of Blood Transfusion Recommendations in Pediatric Critical Care Units
Source: Front Pediatr. 2021 Dec 17;9:800461. doi: 10.3389/fped.2021.800461 (PMC8718763; doi:10.3389/fped.2021.800461)
Supplement: Supplementary file 1 [file Table_1.DOCX]

**Provider interview guide**

| CHANGE AND IMPACT OF CHANGE IN THE ICU  Before we ask questions about blood transfusion, I want to ask you some general questions about how change occurs in your ICU. Are you ready to get started? | |
| --- | --- |
| Core Question | Supplemental Questions |
| 1. Tell me about a [clinical] change in practice that has recently occurred where you work. | - Who proposed the change?  - How was this proposal made?  - Who were the thought leaders who drove the change?  - Who usually has a “say” in terms of change?  - How did you find out about the change? What are the most effective ways people find out about change in your unit?  - How did this change occur in your unit?  - Who enacted the change?  - What methods were used to facilitate change?  - What are the usual barriers to change?  - What types of things help change occur?  - What was your role in the change?  - Do you feel like you have a role/ownership in any changes that occur?  - What went well with this change?  - How could it have been improved?  - What makes change successful or unsuccessful?  - What makes change “stick” in your unit?  - Does the hospital facilitate change/innovation? How? |
| 1. How do you typically respond to change? | - What makes change easier?  - What makes change more difficult?  - Accountability for change (self/peers/supervisor/institution)  - How do you see others respond to change? Receptive to changing practice? |
| 1. If you had a concern or problem related to your work in the unit, who would you discuss it with? | - peers, leadership, administration?  - To what extent do you feel supported?  - Who can you depend on for support?  - Who do others (outside role/group) depend on? |
| 1. How do you know you’ve done a good job at work? | - How do you find out if there are areas where you need to improve?  - What is this experience like?  - How do others get feedback about their work? |
| Thank you very much for that information. Is there anything I haven’t asked about that would be important for me to understand about the working environment in the ICU? | |

| TRANSFUSION DECISION MAKING AND ATTITUDES AROUND RESTRICTIVE TRANSFUSION  One of the aims of the study is to help us understand more about transfusion practices in Pediatric Intensive Care Unit patients. I want to do this by trying to figure out how you think when you’re making the decision to transfuse patients. These questions apply to Red Blood Cells and do not apply to other blood products (platelets, FFP, etc.). | |
| --- | --- |
| Core Question | Supplemental Questions |
| 1. Please describe how you decide to give a patient a blood transfusion.   Nurse modification: From your point of view, what factors seem to influence providers in your unit to give a blood transfusion? | - Physiologic parameters  - Thresholds  - Automatic/automated transfusion?  - Benefits of transfusion?  - Disadvantages of transfusion? |
| 1. Has your approach to transfusion changed over time?   Nurse Modification: From your perspective, have you noticed changes over time in how/when people decide to transfuse? | - Transfuse more or less?  - Why?  - Are you aware of studies that limit or restrict blood transfusion?  - What are your thoughts about these studies (applicable to practice? Of good quality?)? |
| 1. Who else in your unit makes the decision to transfuse patients?   Nurse modification: Who in your unit makes the decision to transfuse patients? | - Residents, fellows, NPs/hospitalists, nurses, subspecialists  - How do others in your unit approach this decision differently than you?  - How does the approach vary? Other criteria or thresholds?  OMIT FOR NURSES: Do you think the approach others have to blood transfusion has changed over time? Why? |
| 1. Do subspecialty groups influence which patients/when patients are transfused? | - In what situations?  - What is their rationale or approach?  - How are disagreements around transfusion resolved? |
| 1. Describe the process of getting blood to the patient once you/someone have/has decided to give a transfusion. | - Process of ordering, administering |
| WATCHING AND WAITING  I am interested to hear your thoughts about how you might manage a patient with borderline Hb by watching & waiting instead of transfusing RBCs. When I say borderline Hb, I’m thinking about those patients when the decision about transfusing might be difficult– where there’s a bit of a grey area. | |
| 1. What would you consider borderline Hb? |  |
| 1. What thought processes guide your decision/(NURSES: the decision) to watch and wait instead of transfusing? | - What influences you to watch and wait? |
| 1. Do any other team members influence whether or not you manage patients with borderline Hb by watching & waiting instead of transfusing RBCs? | - Other clinicians, medical staff including nurses and NPs/residents/fellows, families?  - In what circumstances?  - How do they impact your management? |
| 1. What are the benefits of managing a patient with borderline Hb by watching & waiting instead of transfusing RBCs? | - To self, patients, colleagues, health care organization  - Short term, long term, financial  - What are the disadvantages of watching & waiting?  - to self, patients, colleagues, health care organization  - Short term, long term, financial |
| 1. Does watching & waiting ever conflict with some other goal/objective in terms of patient care? | - anything else that you want to do or achieve?  - Slowing time to recovery, discharge? |
| 1. In what way is managing a patient with borderline Hb by watching & waiting affected by what is going on in the unit or different environmental situations? | - whether the unit is busy  - whether you have to attend to a more urgent clinical issue  - how much time it takes to get RBCs to the unit  - cost, etc. |
| 1. Can you think of situations in which you would be worried about watching and waiting instead of transfusing? | - How does watching and waiting influence your work stress? |
| 1. In comparison to ALL of the other things you do to care for patients, how important do you feel it is to manage a patient with borderline hemoglobin by watching and waiting instead of transfusing RBCs? | - Why? |
| 1. Is there anything I haven’t asked about that would be important for me to understand about red blood cell transfusions in the ICU? |  |
| REVIEW OF TRANSFUSION GUIDELINE, CONTEXTUAL FACTORS AROUND IMPLEMENTATION | |
| 1. What do you think about using guidelines when you care for patients? | - Useful? Constraining?  - Do guidelines affect your professional autonomy? |
| 1. Would a guideline be useful to help guide decision making about transfusion? | - Would you be willing to use a transfusion guideline? Why?    - Would others in your unit be willing to use such a guideline? Why? |
| Interviewer now reviews the new blood transfusion guideline decision tree with the interviewee, briefly running through the points at which the tree “branches” and different recommendations.  Ask: Have you seen this guideline before?  Phone interviews: Did you look at this guideline in advance of our speaking today? | |
| 1. How would this guideline impact your approach to transfusion?   Nurse Modification: How do you think this guideline would impact provider’s approach to blood transfusion in your unit? | - How would it impact patients?  - Advantages to using guideline?  - Disadvantages to using guideline?  - Do you think these guidelines are important? Why? For whom? |
| 1. How well does this guideline “fit” with your current practice/with current practice in your unit? | - What adaptations would be needed? |
| 1. What are the barriers to using this guideline in your unit? | - Features of the guideline itself, logistical challenges  - When in your workflow is the best time to present this information to you?  - What would help people use this guideline in your unit? (workflow, resources/tools needed).  - Who would need to support the guideline in order for it to be used consistently? Other subspecialists? |
| 1. How would you like to learn about a guideline like this? | - What would you like to/need to learn? |
| 1. Is there anything I haven’t asked about that would be important for me to understand about using guidelines or using this transfusion guideline in the ICU? |  |
